# Characterization of a New M13 Metallopeptidase from Deep-Sea *Shewanella* sp. E525-6 and Mechanistic Insight into Its Catalysis

**DOI:** 10.3389/fmicb.2015.01498

**Published:** 2016-01-06

**Authors:** Jin-Yu Yang, Peng Wang, Chun-Yang Li, Sheng Dong, Xiao-Yan Song, Xi-Ying Zhang, Bin-Bin Xie, Bai-Cheng Zhou, Yu-Zhong Zhang, Xiu-Lan Chen

**Affiliations:** ^1^Marine and Agricultural Biotechnology Laboratory, State Key Laboratory of Microbial Technology, Shandong UniversityJinan, China; ^2^Marine Biotechnology Research Center, Shandong UniversityJinan, China

**Keywords:** M13 metallopeptidase, deep-sea bacteria, expression and purification, enzymatic characterization, catalytic mechanism

## Abstract

Bacterial extracellular peptidases are important for bacterial nutrition and organic nitrogen degradation in the ocean. While many peptidases of the M13 family from terrestrial animals and bacteria are studied, there has been no report on M13 peptidases from marine bacteria. Here, we characterized an M13 peptidase, PepS, from the deep-sea sedimentary strain *Shewanella* sp. E525-6, and investigated its substrate specificity and catalytic mechanism. The gene *pepS* cloned from strain E525-6 contains 2085 bp and encodes an M13 metallopeptidase. PepS was expressed in *Escherichia coli* and purified. Among the characterized M13 peptidases, PepS shares the highest sequence identity (47%) with Zmp1 from *Mycobacterium tuberculosis*, indicating that PepS is a new member of the M13 family. PepS had the highest activity at 30°C and pH 8.0. It retained 15% activity at 0°C. Its half life at 40°C was only 4 min. These properties indicate that PepS is a cold-adapted enzyme. The smallest substrate for PepS is pentapeptide, and it is probably unable to cleave peptides of more than 30 residues. PepS prefers to hydrolyze peptide bonds with P1′ hydrophobic residues. Structural and mutational analyses suggested that His531, His535 and Glu592 coordinate the catalytic zinc ion in PepS, Glu532 acts as a nucleophile, and His654 is probably involved in the transition state stabilization. Asp538 and Asp596 can stablize the orientations of His531 and His535, and Arg660 can stablize the orientation of Asp596. These results help in understanding marine bacterial peptidases and organic nitrogen degradation.

## Introduction

In the ocean, High-Molecular-Weight Organic Nitrogen (HMW ON) produced by organisms in the seawater settles and accumulates in the sediment in the form of particulate organic nitrogen (PON) (Brunnegård et al., 2004). Microorganisms play important roles in the degradation and utilization of sedimentary PON. Due to the complexity of PON, it can be supposed that PON degradation needs the coorperation of various proteases and peptidases from sedimentary microorganisms. However, it is still largely unknown how PON is degraded and what kinds of proteases and peptidases are involved in this process in marine sediment. Recently, several proteases from deep-sea bacteria have been reported, such as MCP-01 from *Pseudoalteromonas* sp. SM9913 (Chen et al., 2003), pseudoalterin from *Pseudoalteromonas* sp. CF6-2 (Zhao et al., 2012) and myroilysin from *Myroides* *profundi* D25 (Chen et al., 2009). These bacterial extracellular proteases can hydrolyze HMW ON, such as collagen and elastin, into Low-Molecular-Weight Dissolved Organic Nitrogen (LMW DON), such as peptides and amino acids, and therefore, may play important roles in sedimentary PON degradation (Zhao et al., 2008, 2012; Chen et al., 2009). In addition to proteases that are responsible for HMW ON degradation, bacteria also secrete peptidases to degrade peptides into small oligopeptides and/or amino acids because bacteria generally prefer to absorb oligopeptides of 2–5 amino acid residues and cannot import oligopeptides of more than 35 amino acid residues. Thus far, however, reports on peptidases from marine bacteria are rather few. Therefore, it is still unclear how peptides are degraded into utilizable oligopeptides and/or amino acids for bacterial nutrition in marine sediment.

The M13 family in MEROPS database contains metalloendopeptidases that are restricted to acting on peptides of not more than 40 residues^1^ (Rawlings et al., 2014). The M13 peptidases are wide spread, from bacteria, archaea, fungi to mammals, with the exception of yeast (Bianchetti et al., 2002). To date, more than 4500 sequences in this family have been recorded in MEROPS database. The crystal structures of three enzymes in this family have been solved, that is, neprilysin (NEP) and endothelin-converting enzyme-1 (ECE-1) from human and zinc-dependent metalloprotease-1 (Zmp1) from *Mycobacterium tuberculosis* (Oefner et al., 2000; Schulz et al., 2009; Ferraris et al., 2011). The M13 peptidases of mammals regulate the biological activity of some hormones and peptides and play important roles in blood pressure regulation (NEP) (Ikeda et al., 1999), cardiovascular development (ECE-1) (Xu et al., 1994) and anticipation of hemolytic reaction (KELL) (Lee et al., 2003). Besides the peptidases from mammals, some M13 peptidases from bacteria are also studied. Zmp1 from *M. tuberculosis* may be relevant to the pathogenicity of this bacterium (Master et al., 2008; Johansen et al., 2011; Muttucumaru et al., 2011). The M13 peptidases from *Lactobacillus* spp. can improve the flavor of cheese by hydrolyzing the bitter peptides formed during cheese fermentation (Baankreis et al., 1995; Christensen et al., 2003; Janer et al., 2005). There has been no study on the M13 peptidases from marine bacteria.

*Shewanella* sp. E525-6 (hereafter E525-6) was a strain isolated from a 1190 m deep-sea sediment of South China Sea. In this study, a gene encoding an M13 family peptidase, PepS, was cloned from the genome of E525-6 and expressed in *Escherichia coli*. Recombinant PepS was characterized and its substrate specificity was investigated. Moreover, structural modeling and site-directed mutagenesis were performed to gain insight into the catalytic mechanism of PepS. The results reveal the characteristics of the M13 peptidase PepS from a deep-sea sedimentary bacterium, which sheds light on the degradation of peptides by deep-sea sedimentary bacteria.

## Materials and Methods

### Strains, Plasmids, and Peptides

Strain E525-6 was isolated from the 1190 m deep sea sediment at site 114°35.833′E, 19°24.018′N of South China Sea. The sediment sample was collected during the South China Sea Open Cruise in September, 2008. The 16S rRNA gene sequence of E525-6 was deposited in GenBank under the accession number KT591706. *E. coli* DH5α and *E. coli* BL21(DE3) were purchased from TransGen Biotech (Beijing, China). DH5α was used for gene cloning, and BL21(DE3) was used for gene expression. The vector pET-22b (+) (Novagen, US) was used for the construction of expression vectors. The substrates, including bradykinin, substance P, angiotensin I, neurotensin and oxidized insulin B chain, were purchased from Sigma (US), and big endothelin-1 (big ET-1) from Bachem (Bubendorf, Switzerland). Other peptide substrates, including enkephalin (YGGFM), FSPFR, AAPL and YPLG, were synthesized by Qiangyao Co. Ltd. (Shanghai, China).

### Cloning of the Gene Encoding the M13 Peptidase PepS

The genome DNA of E525-6 was extracted with a bacterial genome extraction kit (Omega, US) according to the manufacturer’s instructions. The obtained genome DNA was subjected to genome sequencing in BGI Tech Solutions Co., Ltd (China). A gene *pepS* encoding an M13 peptidase was deduced from the genome sequence of E525-6. The *pepS* gene was amplified by PCR from the genome DNA of E525-6 with *FastPfu* DNA polymerase (TransGen Biotech, China), and two primers pepS-F/pepS-R (**Table 1**). The amplified gene was verified by sequencing in Beijing Genomics Institute (BGI) Tech Solutions Co., Ltd (China). The sequence of *pepS* was submitted to GenBank under the accession number of KT692986.

**Table 1:** The primers used to amplify *pepS* and its mutants in this study.

Primer name	Primer sequence (5′-3′)
pepS-F	CGCGGATCCGAATAAAGCACTCGTCGG
pepS-R	CCGCTCGAGCCAAATTTTGACTCGTTTTTCG
E532A_a1595c	GCGGTAATTGGACATGCGATGGGCCATGGGTTT
E532A_R	AAACCCATGGCCCATCGCATGTCCAATTACCGC
D538N_g1612a	GATGGGCCATGGGTTTAATGACCAAGGTGCGA
D538N_R	TCGCACCTTGGTCATTAAACCCATGGCCCATC
E592D_a1776t	GCGAACTGACACTAGGTGATAACATTGGTGATCTATCTG
E592D_R	CAGATAGATCACCAATGTTATCACCTAGTGTCAGTTCGC
D596E_t1788g	TAGGTGAAAACATTGGTGAGCTATCTGGTGTGACTATCG
D596E_R	CGATAGTCACACCAGATAGCTCACCAATGTTTTCACCTA
D596A_a1787c	CTAGGTGAAAACATTGGTGCTCTATCTGGTGTGACTATC
D596A_R	GATAGTCACACCAGATAGAGCACCAATGTTTTCACCTAG
H654A_c1960g_a1961c_	TGTGGCCACCGATCCAGCTTCACCCGCTAAGTTC
H654A_R	GAACTTAGCGGGTGAAGCTGGATCGGTGGCCACA
R660K_c1978a_g1979a_c1980g	CATTCACCCGCTAAGTTCAAGTCGCTGGGCGCCC
R660K_R	GGGCGCCCAGCGACTTGAACTTAGCGGGTGAATG


*The underlined are restrict enzyme sites.*

### Expression and Purification of PepS

The obtained *pepS* gene was cloned into the *BamH* I and *Xho* I sites of pET-22b (+) to construct the expression vector pET-22b-*pepS*. The constructed expression vector was transferred into *E. coli* BL21(DE3) for the expression of PepS. The transferred BL21 strain was cultured in Luria-Bertani (LB) medium supplemented with 0.1 mg/ml ampicillin overnight at 37°C. The overnight culture was 100-fold diluted in fresh LB medium with 0.1 mg/ml ampicillin and then cultivated at 37°C to an OD_600_ of 1.0–1.2. The culture was then induced at 15°C for 24 h with 0.2 mM isopropyl-β-D-thiogalactopyranoside (IPTG). After induction, cells in the culture were collected by centrifugation, suspended in the buffer (50 mM Tris-HCl, 100 mM NaCl, 0.5% of glycerol, pH 8.0), and fractured by ultrasonication. The recombinant PepS in the cell extract was purified by affinity chromatography on a His-Tag Ni-affinity column (GE Healthcare, US) and further purified by gel filtration on a superdex G200 column (10 × 300 mm, GE Healthcare, US). The purity of PepS was determined by sodium dodecyl sulfate polyacrylamide gel electrophoresis (SDS-PAGE) as described by Laemmli (1970).

### Site-Directed Mutagenesis to PepS

Using the vector pET-22b-*pepS* as a template, site-directed mutagenesis to PepS was performed with the QuikChange^®^ mutagenesis kit II (Agilent technologies, US) according to the method of QuikChange site-directed mutagenesis experiment (Liu and Naismith, 2008). The primers were designed by QuikChange primer design^2^ (**Table 1**). The mutated genes were transferred into *E. coli* BL21(DE3) to express the mutants. The conditions for mutant culture and protein purification were the same as those of wild-type PepS.

### Protein Determination and Enzyme Assay

Protein concentration was determined by using a Pierce BCA protein assay kit (Thermo Scientific, US) with bovine serum albumin (BSA) as the standard. The activities of PepS or its mutants were measured with the pentapeptide FSPSR as the substrate. The reaction system including 10 μl FSPSR (5 mM) and 30 μl enzyme (0.02 mg/ml) was incubated at 30°C for 10 min. The reaction was stopped by 4 μl of 10% trifluoroacetic acid (TFA). The amount of the product FSP was detected by using a Venusil MP C_18_ liquid chromatography column (Bonna-Agela Technologies, Tianjin, China) on a LC-20AT HPLC (Shimadzu, Japan). The mobile phases contained buffer A (0.1% TFA in ultrapure water) and buffer B (0.1% TFA in acetonitrile). The column was equilibrated with buffer A, and eluted with a linear gradient of 10–35% buffer B in 15 min. The peak area of the product FSP was calculated by integration. One unit was defined as the amount of enzyme for catalysis of FSPFR to produce 1 μmol of FSP in 1 min. The activity of PepS toward oxidized insulin B chain was assayed following the method of Authier et al. (2002).

### N-terminal Amino Acid Sequence Analysis of PepS

Purified PepS in an SDS-PAGE gel was transferred to a Sequi-Blot polyvinylidene difluoride membrance (Bio-Rad, US). Its N-terminal amino acid sequence was determined by Edman degradation with PROCISE491 (Applied Biosystems, US) in Peking University, China.

### Characterization of PepS

To determine the optimum temperature of PepS, the activity of PepS toward FSPSR was measured from 0 to 50°C, and the maximum activity was taken as 100%. To analyze the thermal stability of PepS, PepS was incubated at 35 or 40°C, and the residue activity was measured at 5 min intervals. The activity of the untreated PepS was taken as 100%. To determine the optimum pH of PepS, the activity of PepS in Britton–Robinson buffers ranging from pH 4.0-11.0 was measured, and the maximum activity was taken as 100%. To analyze the effect of NaCl on the activity of PepS, 0.5-3.0 M NaCl was added to the reaction mixture, and then the activity was assayed. The activity of no additional NaCl was taken as 100%. As for the determination of the effect of metal ions on the activity of PepS, 11 metallic compounds were, respectively, added to the reaction mixture at a final concentration of 2 or 4 mM. The mixture was incubated at 4°C for 25 min, and then the activity was assayed. The effect of peptidase inhibitors on the activity of PepS was detected by incubating PepS with each inhibitor at 4°C for 25 min, and then assaying the residual activity. The activity of PepS without any metal ion or inhibitor in the reaction mixture was taken as 100%.

### Analysis of the Substrate Specificity of PepS

The substrate specificity of PepS was investigated by measuring its activities toward bradykinin, substance P, angiotensin I, neurotensin, oxidized insulin B chain, big endothelin-1, enkephalin (YGGFM), FSPFR, AAPL and YPLG. Reactions were all carried out in 10 mM Tris-HCl buffer (pH 8.0) at 30°C for 10 min. The molecular masses of the hydrolytic products were then determined by liquid chromatography-mass spectrometry (LC/MS). The sequences of these released peptides were identified by ExPASy tools^3^ (Gasteiger et al., 2005).

### Homology Modeling and Sequence Analysis

The homology model of PepS was constructed using SWISS-MODEL^4^ with the crystal structure of NEP (1DMT.pdb) as the template (Biasini et al., 2014), and molecular graphics images were produced using PyMOL (DeLano, 2010). Sequence alignment was performed with ClustalW (Chenna et al., 2003) and edited with ESPript (Robert and Gouet, 2014).

### Circular Dichroism Spectra

Circular dichroism (CD) spectra for wild-type PepS and its mutants were recorded at 25°C on a J-810 spectropolarimeter (Jasco, Japan). All the spectra of the proteins at a concentration of 0.2 mg/ml in 10 mM Tris–HCl (pH 8.0) were collected from 250 to 195 nm at a scan speed of 200 nm/min using a quartz cell of 0.1 cm path length.

## Results

### Gene Cloning and Sequence Analysis

**FIGURE 1:**
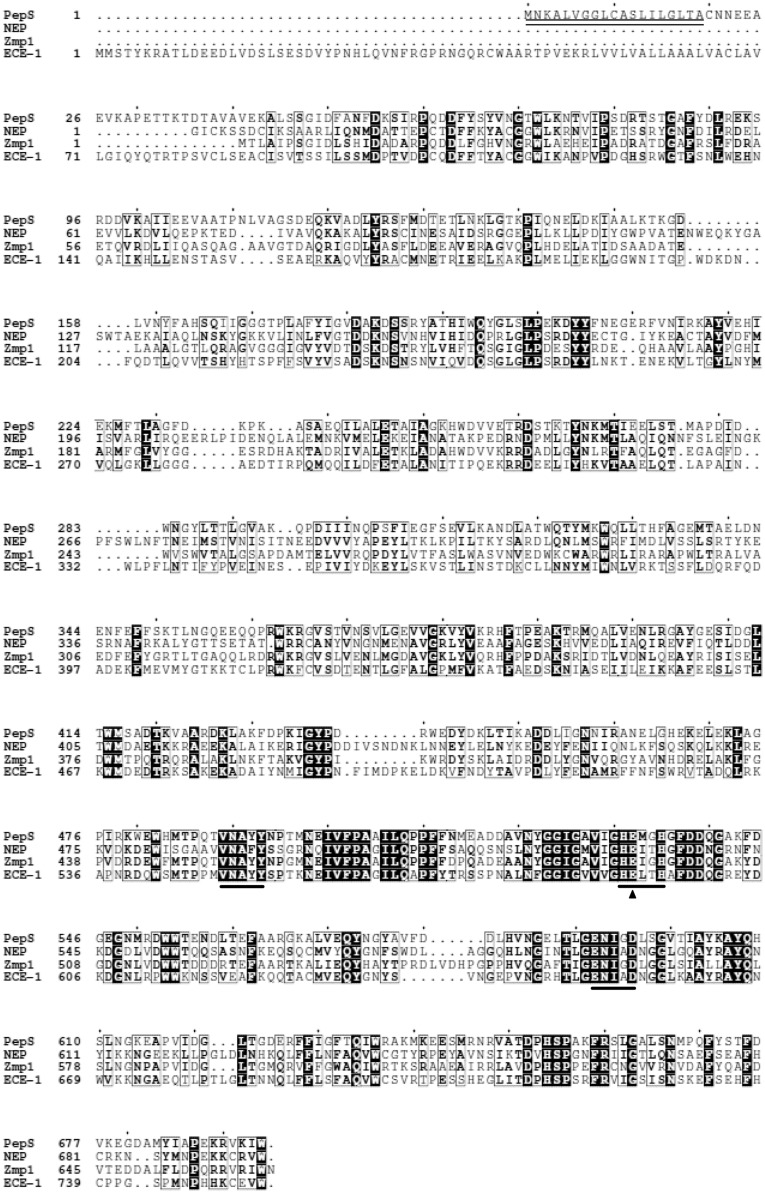
**Sequence alignments of PepS with NEP, Zmp1, and ECE-1 from the M13 family.** Black boxes with white characters indicate residue identity; bold characters indicate residue similarity; black-framed characters indicate similarities between groups of residues. Catalytic Glu532 is labeled with a black triangle. The conserved motifs are labeled by a line, and the predicted signal sequence is labeled by double lines.

The gene *pepS* was cloned from the genome of E525-6. Sequence analysis showed that the ORF of *pepS* contains 2085 bp with an ATG start codon and a TAA stop codon. It encodes a protein (PepS) of 694 amino acid residues with a predicted molecular weight of 77.87 kDa and a predicted isoelectric point (pI) of 5.05. The BLAST analysis suggested that PepS is a Zn-dependent metallopeptidase belonging to the M13 family, containing an M13 catalytic domain (C20 to W694). In addition, it was predicted by SignalP 4.0 Server^5^ (Petersen et al., 2011) that the sequence of PepS contains a signal peptide of 19 amino acid residues (M1 to A19). Sequence alignment of PepS with some M13 peptidases showed that three conserved motifs of the M13 family, HExxH, ENxxD and VNAxx, are also shown in the sequence of PepS (**Figure 1**). These structural characteristics indicate that PepS belongs to the M13 family. Among the characterized M13 peptidases, PepS shows the highest sequence identity with Zmp1 (47%), and higher identities with ECE-1 (31%), and NEP (30%), indicating that PepS is a new member of the M13 family.

### Expression, Purification, and Characterization of PepS

**FIGURE 2:**
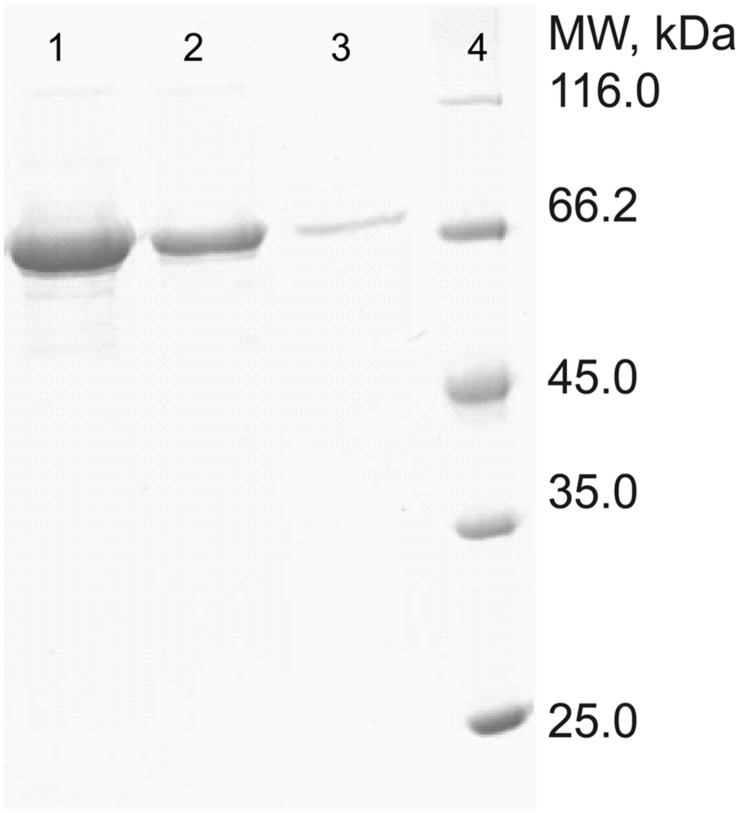
**Sodium dodecyl sulfate polyacrylamide gel electrophoresis analysis of the purified PepS.** Lanes 1–3, purified PepS; lane 4, protein markers.

The full-length gene of PepS was expressed in *E. coli* BL21(DE3), and the recombinant PepS was purified by Ni-NTA affinity chromatography, and sieve chromatography. SDS-PAGE analysis showed that PepS had a high purity after two-step purification (**Figure 2**). N-terminal sequencing of the recombinant PepS showed that the first five residues of the mature enzyme is L17-T-A-C-N21, which indicates that a signal peptide of 16 residues is removed during maturation.

**FIGURE 3:**
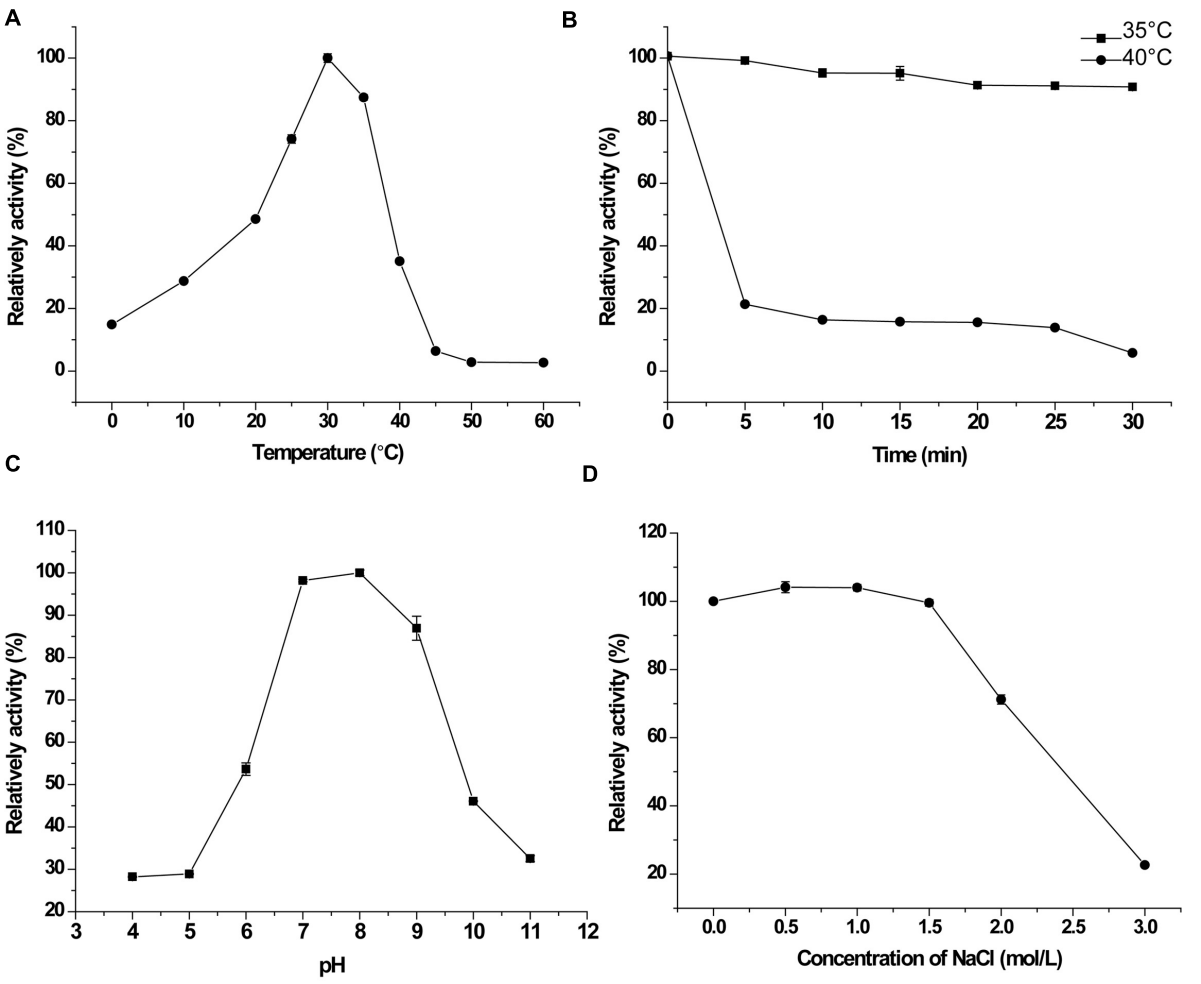
**Characterization of PepS.** **(A)**, Effect of temperature on the activity of PepS toward FSPFR. The highest activity at 30°C was taken as 100%. **(B)**, Stability of PepS at 35°C (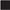) and 40°C (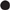). PepS was incubated at 35 or 40°C for different time intervals, and the residual activity was measured at 30°C. The activity of enzyme without heat treatment was taken as 100%. **(C)**, Effect of pH on the activity of PepS toward FSPFR. The activity was measured at 30°C in Britton–Robinson buffers ranging from pH 4.0–11.0. The highest activity at pH 8.0 was taken as 100%. **(D)**, Effect of NaCl on the activity of PepS toward FSPFR. The activity was measured at 30°C in Tris-HCl (pH 8.0, 10 mM) with different concentrations of NaCl. The activity of enzyme in the buffer without NaCl was taken as 100%.

With the pentapeptide FSPFR as the substrate, the optimal temperature for PepS activity was determined to be 30°C. It retained 15% activity at 0°C and no detectable activity at 50°C (**Figure 3A**). PepS retained 95% activity after incubation at 35°C for 30 min, but lost 80% activity after incubation at 40°C for 5 min. The half life of PepS at 40°C was approximately 4 min (**Figure 3B**). These results indicated that PepS is a cold-adapted peptidase. PepS showed the highest activity at pH 8.0, retaining 97% activity at pH 7.0, and 85% at pH 9.0 (**Figure 3C**). PepS activity remained stable in the buffers containing NaCl of 1.5 M or below (**Figure 3D**), suggesting that PepS has good salt tolerance. Among the investigated metal ions, only 2 mM Ca^2+^ and Mg^2+^, had slight activating effects on the activity of PepS, while all the other ions, including K^+^, Sr^2+^, Ba^2+^, Li^+^, Mn^2+^, Co^2+^, Cu^2+^, Zn^2+^, and Sn^2+^, had inhibitory effects on the activity of PepS (**Table 2**). The presence of 10 μM phosphoramidon, a specific inhibitor of Zn^2+^ dependent metallopeptidase, inhibited more than 90% of the activity of PepS, suggesting that PepS is a Zn^2+^ dependent metallopeptidase. Other inhibitors to metallopeptidases, such as 1,10-Phenanthrolin (*o*-P) (2 mM), EDTA (2 mM) and EGTA (2 mM), also strongly inhibited the activity of PepS (**Table 3**).

**Table 2:** Effect of ions on the activity of PepS.

Metal ion	Relatively activity (%)		Metal ion	Relatively activity (%)	
			
	2 mM	4 mM		2 mM	4 mM
Control	100.0^a^	100.0	Li^+^	40.4 ± 2.4	46.4 ± 2.1
Mg^2+^	105.0 ± 2.3	108.0 ± 1.8	Mn^2+^	38.8 ± 1.3	22.6 ± 1.2
Ca^2+^	105.0 ± 1.8	95.0 ± 0.9	Co^2+^	37.1 ± 0.7	56.5 ± 2.3
K^+^	60.4 ± 1.3	52.2 ± 0.5	Cu^2+^	31.0 ± 0.7	40.7 ± 0.6
Sr^2+^	75.5 ± 0.9	25.7 ± 0.8	Zn^2+^	31.0 ± 1.8	16.0 ± 0.4
Ba^2+^	58.4 ± 2.4	76.2 ± 1.4	Sn^2+^	15.1 ± 0.4	0


*^a^The activity of enzyme without any metal ion was taken as control (100%).*

**Table 3:** Effect of inhibitors on the activity of PepS.

Inhibitor	Residual activity (%)
Control	100.0^a^
PMSF (2 mM)	74.1 ± 1.3
EDTA (2 mM)	21.9 ± 0.7
EGTA (2 mM)	14.1 ± 0.9
*o*-P (2 mM)	7.6 ± 0.2
Phosphoramidon (10 μM)	4.3 ± 0.4


*^a^The activity of enzyme without any inhibitor was taken as control (100%).*

### Substrate Specificity of PepS

The specificities of PepS to various substrates were investigated and the cleavage sites of PepS on these peptides were determined by LC-MS (**Supplementary Figures S1–S7**). A comparison of the substrate specificities of PepS and three other M13 peptidases (NEP, ECE-1 and Zmp1) is shown in **Table 4.** PepS had no activity to several tetrapeptides, and could cleave the pentapeptide FSPFR, suggesting that the smallest substrate for PepS is pentapeptide. PepS could effectively hydrolyze oxidized insulin B chain that contains 30 residues, but could not hydrolyze human big ET-1 that contains 38 residues, which implies that PepS may have no activity toward peptides of more than 30 residues. Like NEP, ECE-1 and Zmp1 (Gafford et al., 1983; Skidgel et al., 1984; Johnson et al., 1999; Petrera et al., 2012), PepS efficiently hydrolyzed various hormone substrates, such as bradykinin, substance P, neurotensin and angiotensin I. However, the results also showed that there are some differences in the substrate specificity between PepS and the other three M13 peptidases. On substance P, in addition to the bonds Q6-F7, F7-F8 and G9-L10 that can be cleaved by NEP, ECE-1 and Zmp1 (Skidgel et al., 1984; Johnson et al., 1999; Petrera et al., 2012), PepS could cleave four other bonds, P4-Q5, Q5-Q6, F8-G9 and L10-M11. On oxidized insulin B chain, the peptide bond G8-S9 which has never been reported to be susceptible to M13 peptidases, was found to be cleaved by PepS. In general, PepS prefers to cleave the peptide bonds with hydrophobic or bulky residues at the P1′ site, such as Phe, Val, Ile, and Leu, though it is not strict.

**Table 4:** Comparison of the substrate specificity of PepS with those of NEP, ECE-1, and Zmp1^a^.

Substrate	Sequences	Hydrolyzed peptide bonds			
		
		PepS	NEP	ECE-1	Zmp1
				Johnson et al., 1999	Petrera et al., 2012
	A1APL4	No activity	ND	ND	ND
	Y1PLG4	No activity	ND	ND	ND
	F1SPFR5	P3-F4	ND	ND	ND
Enkephalin	Y1GGFM5	G3-F4	G3-F4 Schwartz et al., 1981	ND	ND
Bradykinin	R1PPGFSPFR9	P7-F8	P7-F8 Gafford et al., 1983	P7-F8	G4-F5 P7-F8
Angiotensin I	D1RVYIHPFHL10	Y4-I5 P7-F8	Y4-I5 P7-F8 Gafford et al., 1983	V3-Y4 Y4-I5 P7-F8	Y4-I5 P7-F8 H9-L10
Substance P	R1PKPQQFFGLM11	P4-Q5 Q5-Q6 Q6-F7 F7-F8 F8-G9 G9-L10 L10-M11	Q6-F7 F7-F8 G9-L10 Skidgel et al., 1984	Q6-F7 F7-F8 G9-L10	Q6-F7 F7-F8 G9-L10
Neurotensin	pE1LYENKPRRPYIL13	L2-Y3 Y11-I12	P10-Y11 Y11-I12 Skidgel et al., 1984	L2-Y3 P10-Y11	P10-Y11 Y11-I12
Oxidized insulin B chain	F1VNQHLC(SO_3_H)GSH10LVEALYLVC(SO_3_H) G20ERGFFYTPKA30	H5-L6 G8-S9 L11-V12 A14-L15 Y16-L17 L17-V18 G23-F24 F24-F25 F25-Y26 T27-P28	H5-L6 H10-L11 L11-V12 A14-L15 L15-Y16 Y16-L17 L17-V18 G23-F24 F24-F25 F25-Y26 Kerr and Kenny, 1974	H10-L11 L11-V12 E13-A14 A14-L15 L15-Y16 Y16-L17 T27-P28	ND
Big ET-1 (human)	C1SCSSLMDKE10CVYFCHLDII20WVNTPEHVVP30YGLGSPRS38 (Disulfide bonds between C1 and C15/C3 and C11)	No activity	ND	W21-V22 Turner and Murphy, 1996	ND


*ND, no detection.*

*^a^The substrate specificities of NEP, ECE-1, and Zmp1 were cited from references as shown in the table.*

### Key Residues for the Catalysis of PepS

**FIGURE 4:**
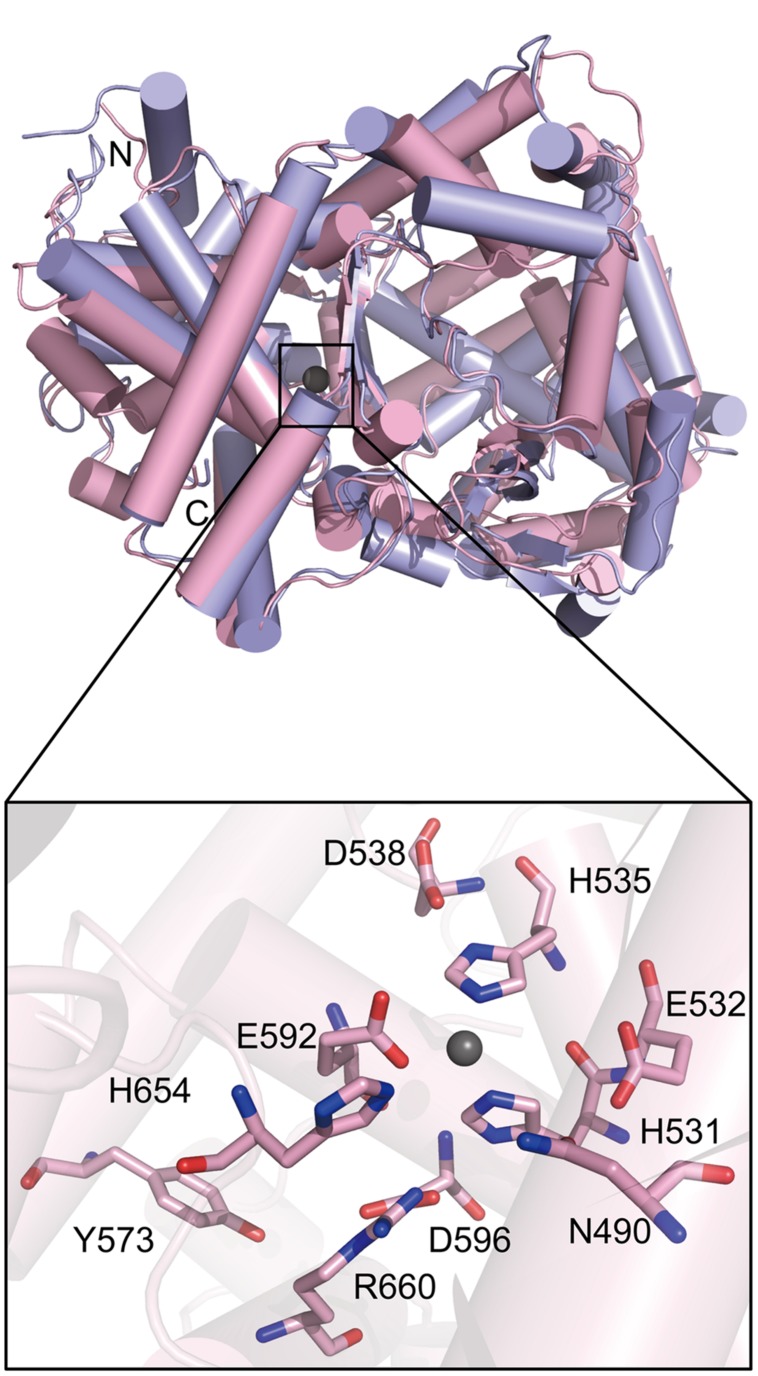
**Ribbon plot models of PepS and NEP and the conformation of the active center of PepS.** PepS is shown in pink, and NEP is shown in light purple. The zinc ion is shown with a gray ball. N, the amino terminus. C, the carboxyl terminus.

To investigate the catalytic mechanism of PepS, we tried to obtain the crystal structure of PepS, but failed. Instead, we obtained a structural homology model of PepS using Swiss-Model^4^ with the structure of NEP (PDB ID: 1DMT) as a template. The model of PepS presents a similar overall structure to NEP (**Figure 4**). An analysis of the conformation of the active center of PepS suggested that the zinc ion of PepS is coordinated by His531 and His535 in the motif HExxH, as well as Glu592 (**Figure 4**).

**FIGURE 5:**
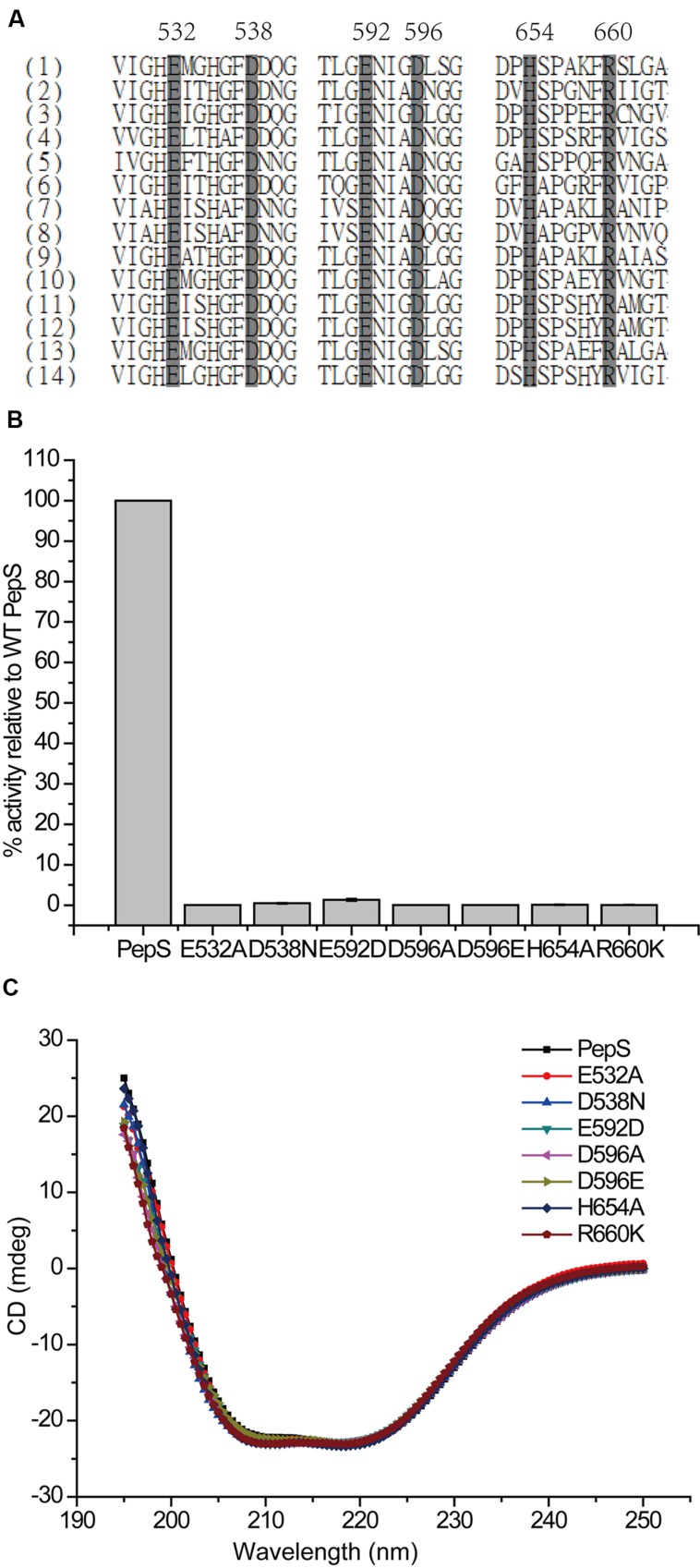
**Analysis of the key residues for the catalysis of PepS.** **(A)** A multiple sequence alignment of M13 peptidases (1) PepS (*Shewanella* sp. E525-6); (2) NEP (*Homo sapiens*); (3) Zmp1 (*Mycobacterium tuberculosis*); (4) ECE-1 (*Homo sapiens*); (5) PHEX peptidase (*Mus musculus*); (6) Nep2 peptidase (*Bombyx mori*); (7) oligopeptidase O1 (*Lactococcus lactis*); (8) oligopeptidase O2 (*Lactobacillus helveticus*); (9) Zmp1 peptidase (*Xanthomonas campestris*); (10) Zmp1 peptidase (*Idiomarina loihiensis*); (11) Zmp1 peptidase (*Shewanella* sp. MR-4); (12) Zmp1 peptidase (*Shewanella* sp. W3-18-1); (13) Zmp1 peptidase (*Shewanella oneidensis*); (14) Zmp1 peptidase (*Pseudoalteromonas* sp. BSi20480). Gray boxes with black characters indicate residue highly reserved, and the numbers of PepS are labeled. **(B)** The relative activities of wild-type PepS and its mutants. The activity of wild-type PepS was taken as 100%. **(C)** Circular dichroism spectra of wild-type PepS and its mutants.

To determine the residues important for the catalysis of PepS, based on sequence alignment and structural analysis (**Figures 4** and **5A**), some conserved residues at or near the active center of PepS were selected for site-directed mutation. Glu532 is in the motif HExxH of PepS, and the equivalents of Glu532 in other M13 peptidases are supposed to act as a nucleophile during catalysis (Devault et al., 1988a; Oefner et al., 2000; Schulz et al., 2009; Tallant et al., 2010; Ferraris et al., 2011). Mutation of Glu532 to Ala abolished the activity of PepS to FSPFR completely (**Figure 5B**), which indicated that Glu532 is essential for the catalysis of PepS, and most likely functions as a nucleophile. Glu592 is a coordinating residue of the zinc ion in PepS. The activity of mutant E592D to FSPFR completely lost although Glu and Asp are both acidic amino acids (**Figure 5B**), indicating that Glu592 plays an important role in PepS catalysis. According to the structural model of PepS, the side chain of Asp538 can form a hydrogen bond with His535, and the side chain of Asp596 can form a hydrogen bond with His531. Both His535 and His531 are in the motif HExxH. In NEP, the side chain of Asp591 (counterpart of Asp538 of PepS) and Asp651 (counterpart of Asp596 of PepS) are thought to form triads with the coordinating histidine residues and take part in placing zinc ion in a suitable position for catalysis (Le Moual et al., 1994). Mutants of both D538N and D596A completely lost their activities (**Figure 5A**), which suggests that Asp538 and Asp596 in PepS might play similar roles as their counterparts in NEP in helping to fasten the zinc ion by forming hydrogen bonds with the coordinating His residues. Structural analysis indicated that Arg660 might form a salt bridge with Asp596. Although Arg and Lys are both basic residues, mutation of R660 to Lys caused a complete loss of the catalytic activity of PepS (**Figure 5B**), indicating that Arg660 is also important for PepS catalysis by stablizing the orientation of Asp596 via a salt bridge. In addition, the mutant H654A also presented little catalytic activity to FSPFR (**Figure 5B**). In NEP, His711 (counterpart of His654 of PepS) is suggested to stabilize the transition state (Dion et al., 1993). Therefore, His654 in PepS may have a similar role to stabilize the transition state. Moreover, CD spectral analysis showed that these site-directed mutations caused little changes in PepS structure (**Figure 5C**), indicating that the activity loss of these mutants was caused by residue substitution rather than structural change. Taken together, these results indicate that Glu532, Asp538, Asp592, Asp596, His654, and Arg660 are key residues for the catalysis of PepS. These residues are high conserved among the M13 peptidases (**Figure 5A**), suggesting that PepS and other M13 peptidases are most likely to have the same catalytic mechanism.

## Discussion

The M13 family peptidases are widely distributed, ranging from mammals to bacteria (Bianchetti et al., 2002). While previous research have focused on the M13 peptidases from terrestrial organisms, there has been no study on enzymes of this family from marine organisms. Up to date, a large number of peptidase sequences of the M13 family have been deduced from the genomes of *Shewanella* spp. however, none of these sequences has been characterized. In this study, an M13 family peptidase, PepS, from a marine bacterium *Shewanella* sp. E525-6 was characterized. PepS shares 30% sequence identity with NEP (Oefner et al., 2000), the prototype of the M13 family. Among the characterized M13 peptidases, PepS shows the highest sequence identity (47%) with Zmp1 from *Mycobacterium tuberculosis* (Ferraris et al., 2011). The M13 peptidases usually have three typical conserved motifs in their sequences, the zinc-metallopeptidase motif HExxH, the specific active center motif ENxxD, and the substrate binding motif VNAxx (Oefner et al., 2000). Sequence analysis showed that PepS also have these three conserved motifs in its sequence. In addition, the substrate specificity of PepS is generally similar to those of the other M13 peptidases, such as NEP, ECE-1 and Zmp1 (Gafford et al., 1983; Skidgel et al., 1984; Johnson et al., 1999; Petrera et al., 2012). These characters show that PepS is a new member of the M13 family.

E525-6 was isolated from a 1190 m deep-sea sediment of South China Sea. Deep sea sediment is an extreme habitat for bacteria, with low temperature, high salt concentration and high pressure. Bacterial enzymes from deep-sea environment may display distinct features compared with their homologs from other environments. PepS displayed a low optimum temperature of 30°C, an optimum pH of 8.0, and remained stable upon 1.5 M NaCl, indicating that it is a cold-adapted, basic-preferred and salt-tolerant M13 peptidase. These features enable PepS to adapt to the extreme deep-sea environment and play a role in LMW DON degradation in deep-sea sediment.

The M13 peptidases are only active on substrates which are probably restricted to peptides no more than 40 residues (Rawlings et al., 2014). Because most of the characterized M13 peptidases are from animals, the tested substrates for these enzymes are mainly various hormone substrates, such as bradykinin, substance P, neurotensin and angiotensin I. Structural analysis shows that the residues forming the S1′ subsite of the M13 peptidases are mainly hydrophobic and conserved, and the hydrophobic S1′ pocket favorably accommodates a substrate with a bulky and hydrophobic P1′ side chain (Oefner et al., 2000; Schulz et al., 2009; Ferraris et al., 2011). Therefore, the M13 peptidases prefer to hydrolyze peptide bonds having a P1′ residue with a hydrophobic bulky side chain (Gafford et al., 1983; Skidgel et al., 1984; Johnson et al., 1999; Petrera et al., 2012). Consistent with this, sequence alignment shows that the residues forming the S1′ subsite of PepS are all hydrophobic resides (Phe88, Ile506, Phe511, Ala527, Val528, Ile635, and Trp636), which makes PepS have a similar substrate specificity with other M13 peptidases, preferring to hydrolyze peptide bonds with a P1′ residue with a hydrophobic bulky side chain, such as Phe, Val, Ile, and Leu. However, in addition to the peptide bonds cleaved by other reported M13 peptidases (Skidgel et al., 1984; Johnson et al., 1999; Carson et al., 2002; Petrera et al., 2012), we found that PepS also could hydrolyze the bonds Q5–Q6 and F8-G9 on substance P, and the bond G8-S9 on oxidized insulin B chain. These three bonds are first found to be cleaved by an M13 peptidase on the referred substrates, to our knowledge. It also indicates that the substrate specificity of PepS on P1′ site is not strict to residues with a hydrophobic bulky side chain.

The structures of three M13 peptidases, NEP, ECE-1 and Zmp1 have been solved (Oefner et al., 2000; Schulz et al., 2009; Ferraris et al., 2011). Based on structural and biochemical analyses, the catalytic mechanism of NEP has been revealed (Devault et al., 1988a,b; Le Moual et al., 1991; Dion et al., 1993; Le Moual et al., 1994; Oefner et al., 2000). In the catalytic center, the catalytic zinc ion is coordinated by two histidine residues in the conserved motif HExxH, one glutamic acid residue and one water molecule. When a substrate molecule enters the catalytic cavity, the glutamic acid residue in the motif HExxH functions as a nucleophile to attack the water molecule coordinating the zinc ion, which initiates the catalytic process (Oefner et al., 2000). Based on the structural homology model, PepS has a similar conformation of the active center with NEP, suggesting that PepS may have the same catalytic mechanism as NEP. Structural and biochemical analysis on PepS indicated that His531, His535 and Glu592 form a triad to coordinate the zinc ion, and Glu532 acts as a nucleophile to trigger the catalytic process. In addition, Asp538, Asp596, Arg660 and His654 are also important for the catalysis of PepS. Asp538 and Asp596 stablize the orientation of the histidine residues coordinating the zinc ion via hydrogen bonds, Arg660 stablizes the orientation of Asp596 via a salt bridge, and His654 may be involved in the stabilization of the transition state.

In summary, this study characterized an M13 peptidase from a deep-sea sedimentary bacterium E525-6, and its substrate specificity and catalytic mechanism were also studied. This represents the first report on an M13 peptidase from a marine bacterium, to our knowledge. The results will be helpful for our understanding of the degradation and utilization of organic nitrogen in deep sea sediments.

## Author Contributions

J-YY designed, conducted and composed the paper. PW made the structure modeling. C-YL analyzed the modeling structure and revised the paper. SD offered the strain. X-YS, X-YZ, B-BX, B-CZ, and Y-ZZ analyzed the data. X-LC instructed the study and revised the paper.

## Conflict of Interest Statement

The authors declare that the research was conducted in the absence of any commercial or financial relationships that could be construed as a potential conflict of interest.

## Footnotes

1 http://merops.sanger.ac.uk/cgi-bin/famsum?family=M13

2 http://www.genomics.agilent.com/primerDesignProgram.jsp

3 http://web.expasy.org/findpept/

4 http://swissmodel.expasy.org/

5 http://www.cbs.dtu.dk/services/SignalP-4.0/
