# Supplementary material for: Characterization of a New M13 Metallopeptidase from Deep-Sea Shewanella sp. E525-6 and Mechanistic Insight into Its Catalysis
Source: Front Microbiol. 2016 Jan 6;6:1498. doi: 10.3389/fmicb.2015.01498 (PMC4701951; doi:10.3389/fmicb.2015.01498)
Supplement: Supplementary file 1 [file Data_Sheet_1.DOC]

FSP

FSPFR

**Fig S1. LC/MS analysis of the cleave site of FSPFR by PepS.**

FM

YGGFM

**Fig S2. LC/MS analysis of the cleave site of enkephalin (Y1GGFM5)** **by PepS.**

RPPGFSP

RPPGFSPFR

**Fig S3. LC/MS analysis of the cleave site of bradykinin (R1PPGFSPFR9) by PepS.**

DRVYIHP

FHL

IHPFHL

DRVYIHPFHL

**Fig S4. LC/MS analysis of the cleave site of angiotensin I (D1RVYIHPFHL10) by PepS.**

RPKPQQF

RPKPQQF

LM

FG

FGLM

FFG

QQFFGL

FF

RPKPQ

**Fig S5. LC/MS analysis of the cleave site of substance P (R1PKPQQFFGLM11) by PepS.**

YENKPRRPYIL

IL

pE1LYENKPRRPYIL13

**Fig S6. LC/MS analysis of the cleave site of neurotensin (pE1LYENKPRRPYIL13) by PepS.**

FYTPKA

LY

|  | | |  |  |  |  |
| --- | --- | --- | --- | --- | --- | --- |
|  | | |  |  |  |  |
|  |  |  |  | | | |

LC(SO_3_H)GSHL

FVNQHLC(SO_3_H)GSHL

FFYTPKA

LVC(SO_3_H)GERGF

VEALY

SHLVEALYL

VC(SO_3_H)GERGFFYTPKA

FF

LYL

FYT

**Fig S7. LC/MS analysis of the cleave site of oxidized insulin B chain by PepS.**

Oxidized insulin B chain (F1VNQHLC(SO_3_H)GSH10LVEALYLVC(SO_3_H) G20ERGFFYTPKA30)
